# Effects of interspinous spacers on lumbar degenerative disease

**DOI:** 10.3892/etm.2013.894

**Published:** 2013-01-15

**Authors:** DONG ZHOU, LU-MING NONG, RUI DU, GONG-MING GAO, YU-QING JIANG, NAN-WEI XU

**Affiliations:** Department of Orthopaedics, Changzhou No. 2 Hospital, Jiangshu, P.R. China

**Keywords:** lumbar degenerative disease, In-space, interspinous dynamic stabilisation

## Abstract

The present study aimed to evaluate the early effects of interspinous spacers on lumbar degenerative disease. The clinical outcomes of 23 patients with lumbar degenerative disease, treated using interspinous spacer implantation alone or combined with posterior lumbar fusion, were retrospectively studied and assessed with a visual analogue scale (VAS) and the Oswestry Disability Index (ODI). Pre-operative and post-operative interspinous distance, disc space height, foraminal width and height and segmental lordosis were determined. The early effects and complications associated with the interspinous spacers were recorded. The surgical procedures performed with the in-space treatment were easy and minimally invasive. The VAS scores and ODI were improved post-operatively compared with pre-operatively. Significant changes in the interspinous distance, disc space height, foraminal width and height and segmental lordosis were noted. In-space treatment for degenerative lumbar disease is easy and safe, with good early effects. The in-space system provides an alternative treatment for lumbar degenerative disease.

## Introduction

Lumbar degenerative disease is the most common cause of lower back pain (1–3). The conventional treatment for this disease is spinal fusion (4), a method that was developed in the early 20th century and which has been widely used in the treatment of lumbar degenerative disease. Lumbar interbody fusion functions by fixing the lesioned segments together via fusion, eliminating intercalated disc degeneration and vertebral pathological motion, changing the biological and mechanical environment and relieving osphyalgia (4,5). However, a number of clinical treatments show that fusion surgery has no evident advantages compared with non-operative treatments (6,7). By contrast, fusion fixing may defunctionalise the corresponding spinal segments, which may lead to the load stress being concentrated in the adjacent segments and abnormal activity levels increasing, thus speeding up the degeneration of the adjacent segments (8–11). With the extensive developments in lumbar fixed fusion and the long-term follow-up of patients, the aggravated degeneration of the adjacent segments subsequent to fusion gradually caught the attention of spinal surgery doctors (12,13). As a result, dynamic stabilisation (non-fusion technology) was proposed as a new treatment.

Non-fusion dynamic stabilisation was introduced for the treatment of spinal degenerative disease in a near-normal spinal physiological environment. Dynamic stabilisation only fixes the lumbar spine and does not fuse it. The normal stress transmission mode of the motion segments may be recovered by changing the load-bearing functions of the motion segments and by limiting the range of the unstable motion segments. In this way, the intervertebral discs that degenerate in middle age may be spontaneously repaired, thereby relieving pain (14). Dynamic stabilisation allows the lesion segments to retain certain abilities and reduces the effects of stress and movement of the adjacent segment to avoid or delay adjacent segment degeneration (15). Although the effectiveness and indications of dynamic stabilisation have yet to be further studied, the concepts and methods behind dynamic stabilisation have already been accepted by the majority of spinal surgery doctors. Dynamic stabilisation has shown good primary clinical outcomes and increased clinical applications in artificial intervertebral discs, prosthetic disc nuclei, the elastic pedicle system and interspinous process fixation system. Further clinical and basic investigations are currently being processed (16–18).

In previous years, the clinical applications of the interspinous process fixation system have increased, with satisfactory preliminary clinical reports at home and abroad (19–21). In-space belongs to the dynamic interspinous process fixation system and is a novel development and design by Synthes (West Chester, PA, USA) for use in clinical applications. Compared with other systems, in-space is a minimally invasive surgery that aims to restrict lower back pain caused by excessive stretching of the lumbar vertebrae. Therefore, in-space is considered to perform satisfactorily in the treatment and prevention of lumbar degeneration and instability (22). In the present study, the effects of interspinous spacers in lumbar degenerative disease were investigated.

## Patients and methods

### General data

Of the 23 patients with lumbar degenerative disease, 13 were male and 10 were female, with an age range of 20–78 years old (mean, 43.8). The patients suffered from varying degrees of lumbar and back pain caused by excessive lumbar stretches. These patients experienced temporary relief in the flexed position, however, dynamic X-ray positioning indicated lumbar instability. The present study recorded 3 cases of lumbar lateral recess stenosis (L4/5), 12 cases of lumbar disc herniation (10 cases of L4/5 and 2 cases of L3/4) and 2 cases of adjacent segmental slippage (1 case of L4/5 slippage with L3/4 instability and 1 case of L5S1 slippage with L4/5 instability). Subsequent to 3 months of normal conservative treatments, the physical conditions of these patients revealed no severe osteoporosis, psychological disorders or surgical taboos. The study was approved by the ethics committee of Nanjing Medical University, Nanjing, China and written informed patient consent was obtained from the patient.

### Surgical methods

Patients treated with in-space alone were subjected to surgery under local anaesthesia in the intraoperative prone position, with an auxiliary abdominal pad to increase interspinous spacing. Anatomical landmarks were marked on the surface of the skin. Small joints and interspinous ligaments were narcotised in the AP position with lateral percutaneous anaesthesia. A guidewire was placed into the horizontal or vertical incision approximately 2 cm in the lateral direction, through interspinous under the guidance of the perspective and always parallel to the coronal section. Fixing the guidewire subsequently and the interspinous spacer was placed in it. The expected opening (the inferior endplate of the superior vertebrae parallel to the superior endplate of the inferior vertebrae) was followed with a matched implant placed into the sleeve, attached to the margin of the upper and lower spinous processes. An appropriate implant was inserted and its flank was fully unfolded in a good position.

The cases with the clinical symptoms of herniated discs and lumbar spinal osseous stenosis were subjected to a lumbar disc excision using a minimally invasive disc scope and percutaneous in-space implant following full decompression of the nerve root canal. The cases with adjacent segmental slippage that required fixation and fusion of the adjacent segments were first provided with pedicle screw implantations, with interspinous decompression and fusion in the corresponding segments, followed by in-space implantation and finally pedicle system longitudinal rod implantation.

### Effect evaluation

Pre-operative and post-operative pain assessments were conducted using the visual analogue scale (VAS, pain scores of 0 to 10) and the Oswestry Disability Index (ODI).

### Imaging evaluation

Pre-operative (1 day) and post-operative (2 week and 1, 3, 6, 12 and 18 month) lateral views were prepared in neutral, flexion and extension positions, as well as anteroposterior views of post-operative lumbar roentgenograms. Whether the in-space system shifted or not was determined by observing and measuring the distance between spinous processes, the width and height of the intervertebral foramen, the height of the intervertebral anterior and posterior margins, the lumbar segmental lordosis angle and the degree of segmental mobility. After >6 months, patients without lumbar disc excision underwent post-operative rechecks using MRI to show the recovery of the disc lesions in the in-space implanted segments and adjacent segments.

### Statistical analysis

Pre-operative and post-operative pain VAS and ODI scores are expressed as mean ± standard deviation. A paired t-test was employed through statistical software SPSS 11.0. P<0.05 was considered to indicate a statistically significant difference.

## Results

### Clinical effects

The surgeries were successfully performed in all cases. The average implant time when using in-space was 19±4 min. Only minimal bleeding occurred and the stitches were removed subsequent to the wounds healing fully.

Final follow-up results were recorded at 6 months, although the total follow-up period ranged from 6 to 18 months, with a mean of 11±2.9 months. Varying degrees of improvement were identified in the post-operative symptoms. The VAS pain scores at 1 day pre-operation, 2 weeks post-operation and the final follow-up were 7.8±2.1, 2.9±1.3 and 1.5±0.7 out of 10, respectively. The VAS pain scores subsequent to the surgery were lower than those observed prior to the surgery (P<0.01). The VAS pain score at the final follow-up was also lower than that at 1 week post-operation (P<0.05). The ODI scores at 1 day post-operation, 2 weeks post-operationu and the final follow-up were 87.3±9.1, 54.8±6.7 and 10.6±2.1%, respectively. The ODI score at the final follow-up was significantly lower than that at 1 day pre-operation and 2 weeks post-operation (P<0.01). The ODI score at 2 weeks post-operation was also lower than that at post-operation (P<0.05).

### Imaging evaluation

**Figure 1.**
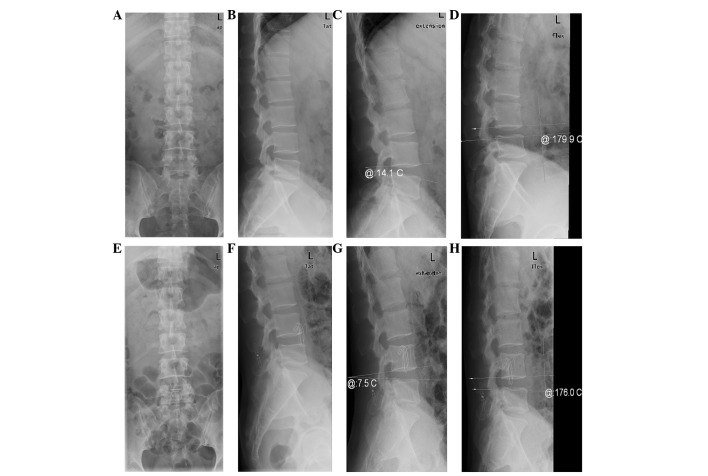
X-rays of (A) a pre-operative neutral position and (B) a pre-operative lateral position to measure the distance between spinous processes, the width and height of the intervertebral foramen, the height of the intervertebral anterior and posterior margins, as well as the lumbar segmental lordosis angle. (C) Pre-operative hyperextension and (D) hyperflexion positions showed that the segments mobilities were >11° and enabled measurements and comparisons of the corresponding distances. (E) In-space position was observed in the post-operative neutral position whether the lateral wings had been completely opened or crimped. (F) Post-operative lateral position showed increases in the distance between spinous processes, the width and height of the intervertebral foramen and the height of the intervertebral posterior margin, as well as decreases in the height of the intervertebral anterior margin and the lumbar segmental lordosis angle. (G) Pre-operative hyperextension and (H) hyperflexion positions showed that the mobilities of the abnormal segments were significantly decreased.

At 1 day pre-operation, 2 weeks post-operation and the final follow-up, the distances between the spines were 4.2±0.5, 9.2±1.1 and 9.1±1.2 mm, respectively. Statistically significant differences were identified between the results at 2 weeks post-operation and 1 day pre-operation as well as between the results at the final follow-up and 1 day pre-operation (P>0.05). However, no significant difference was identified between the results at 2 weeks post-operation and the final follow-up (P<0.05). The widths and heights of the intervertebral foramen were 8.5±1.1 and 18.7±2.1 mm at 1 day pre-operation, 10.8±1.3 and 21.4±2.3 mm at 1 week post-operation and 10.9±1.4 and 21.1±2.5 mm at the final follow-up, respectively. Similarly, statistically significant differences were identified between the results at 2 weeks post-operation and 1 day pre-operation as well as between the results at the final follow-up and 1 day pre-operation (P>0.05). However, no statistically significant differences were identified between the results at 2 weeks post-operation and the final follow-up (P<0.05). The heights of the intervertebral anterior and posterior margins were 13.6±1.5 and 7.7±0.9 mm at 1 day pre-operation, 12.7 ±1.3 and 11.3±1.4 mm at 2 weeks post-operation and 12.9±1.5 and 11.1±1.6 mm at the final follow-up, respectively. The anterior and posterior margin heights were significantly higher at 2 weeks post-operation and significantly lower at the final follow-up compared with those at 1 day pre-operation (P>0.05). However, no statistically significant differences were identified between the results at 2 weeks post-operation and the final follow-up (P<0.05). The lumbar segmental lordosis angle and segmental mobility were 14.4±1.7 and 21.6±5.8° at 1 day pre-operation, 7.5±1.2 and 6.2±1.6° at 2 weeks post-operation and 7.9±1.4 and 6.8±1.5° at the final follow-up, respectively. A significant improvement in the results was noted at 2 weeks post-operation and the final follow-up compared with the results at 1 day pre-operation (P>0.05). However, no statistically significant differences were identified between the results at 2 weeks post-operation and the final follow-up (P<0.05; Table I; Fig. 1).

**Figure 2.**
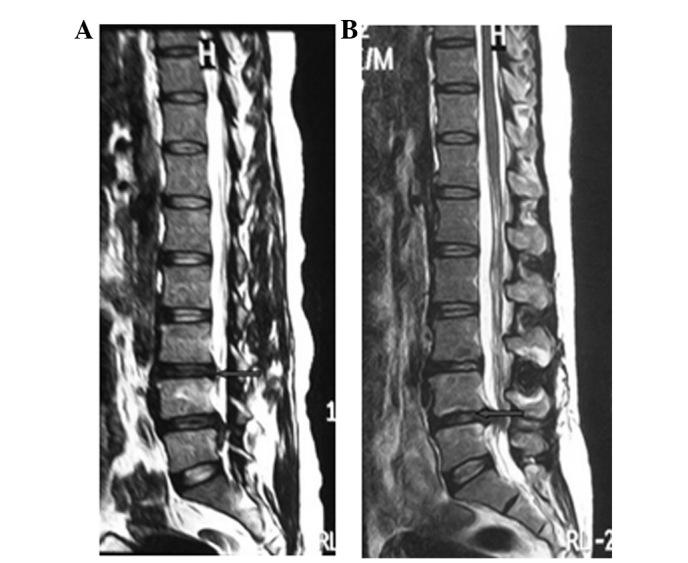
(A) Pre-operative MRI and (B) post-operative MRI at 6 months showed signal changes of hydration in L3/4. (A) Since a pre-operative herniated disc was indicated in L3/4 and L4/5, L4/5 underwent excision of the intervertebral disc and L3/4 underwent in-space implantation. T2 images showed that the hydration signals of L3/4 and L4/5 were poorer than those of other intercalated discs. (B) Post-operative rechecks at 6 months suggested significant improvements in the hydration signals of L3/4. Although L4/5 suffered a herniated disk, it was considered a post-operative recurrence.

No shifts in the in-space system or spinous fractures were observed in any follow-up cases. After >6 months, patients without lumbar disc excision underwent a post-operative recheck by MRI. The results showed that the disc hydration signals of the treated and adjacent segments at 6 months post-operation were superior to those at day 1 pre-operation (Fig. 2).

## Discussion

In the present study, the in-space system was mainly composed of two polyetheretherketone-based cylinders connected by a titanium alloy rod. The upper wing of the titanium alloy was opened through the central mechanical rotating device to prevent lateral sliding. A ‘floating’ device was formed in the interspinous process to increase the distance between spinous processes and the intervertebral foramen. The device was able to restrict excessive stretching in the implanted segment, reduce pressure stress in the interspinous process and zygapophysial joints and retain a certain range of motility in the corresponding segment. As a result, excessive movements that accelerate degeneration and instability are avoided, waist pain caused by dynamic stenosis of the intervertebral foramen is alleviated and the effect that fusion has on the adjacent segments is prevented (23,24). Diaz *et al*(25) showed that the minimally invasive in-space system was able to effectively prevent and treat lumbar spinal stenosis and neurogenic claudication caused by lumbar degeneration, as well as reducing adjacent segment degeneration and the lower back pain caused by it. The in-space system may also be used to treat the following lumbar degenerative diseases: i) central, lateral and intervertebral lumbar spinal stenosis, accompanied by one-sided leg, hip and groin pains that are relieved by flexion; ii) herniated discs, accompanied by lower back pain; iii) facet joint symptoms caused by inflammation on the articular surface; iv) degenerative spondylolisthesis below the first degree with excessive lordosis; v) degenerative disc disease with sacral migration; and vi) interspinous pains caused by Baastrup’s syndrome (spinous process consistent).

The patients in the present study suffered from varying degrees of lumbar spine instabilities. Imaging indicated dynamic spinal stenosis, which is clinically characterised by lower back pain or radiating pains in the hyperextended position. The patients experienced relief from this condition in the flexed position. The elderly patients underwent routine pre-operative bone mineral density tests, after which patients with tests showing two or more standard deviations less than the normal were not recommended in-space implantation. The stability of in-space depended on the integrity of certain elements, including the supraspinal ligament, vertebral plate, spinous process and zygapophysial joints. Therefore, considering that the majority of patients had herniated discs or spinal stenosis, lumbar disc excision or spinal expansion was undertaken using minimal invasion prior to in-space implantation to reduce any bone destruction or ligament/muscle injuries. In-space should be implanted near the ventral side as the basal section of the spinous process provides stronger support. This method results in less trauma and simple surgeries. Compared with other spinous dynamic stabilising devices, the simple in-space system implantation used in the present study only required local anaesthesia, took a short time and produced minimal intraoperative bleeding. The mean surgical time for the implantation system was 19±4 min, which may be further shortened in the future as further experience leads to improved technical skills. The system had almost no learning curve period, with no special requirements in surgical corollary equipment, with the exception of the C arm machine. Hence, this technology may be rapidly spread and the indications are easily understood. This machine was safely and easily used. In-space system implantation may cause minor damage to the normal structure of the posterior spines, only causing injuries to the interspinous ligaments but not interfering with the canalis vertebralis or damaging the nerve root. During the revision surgery, the surgery was safe and easy as the first surgery retained the posterior spinal structure. No marked peri-operative complications were observed. During the follow-up, no system shifting or loosening and no spinous fractures were observed. The heights and widths of the intervertebral foramen were larger subsequent to the surgery than prior to it, particularly in the hyperextended position. Therefore, patients with spinal stenosis caused by hyperextension should be provided with in-space treatment with a moderate opening of implanted gap to restrict the back extension of the surgical segments, expand the canalis vertebralis and nerve root canal to a limited extent and effectively prevent spinal stenosis. In the present study, the segment mobilities subsequent to the surgery were markedly less than prior to the surgery. Therefore, patients with segment instabilities should be provided with in-space implantation to effectively prevent the excessive activities and sliding of the segments. Also, the height of the interspinous posterior margin and the distance between spinous processes were significantly larger subsequent to the surgery. The patients without lumbar disc excision underwent MRIs at 12 months post-operation and the disc hydration signals of the treated and adjacent segments were observed to be higher at 12 months post-operation compared with at 1 day pre-operation. This finding suggests that patients with herniated discs should be provided with in-space implantation to effectively alleviate the pressure in the intervertebral space and prevent any significant increase in the stress on the adjacent disc. Consequently, a recurrent herniated nucleus pulposus or secondary herniated adjacent disc may be avoided.

In sumary, the research direction and goals of clinical treatments for lumbar pain should include maintaining the stability of the reconstruction following lumbar degeneration and instability, keeping normal intervertebral mobility in the treated segments and reducing the complications that may be caused by further treatments. Using the in-space interspinous process distraction system alone or in combination with fixation and fusion methods in the treatment of lumbar degenerative disease is a simple and safe treatment, with a good curative effect observed in the initial follow-up. Therefore, the in-space system is a new treatment option for lumbar degenerative diseases.

## Figures and Tables

**Table I.** Pre- and post-operative distances between the spines, the widths and heights of the intervertebral foramen, the height of the intervertebral anterior and posterior margins, as well as the lumbar segmental lordosis angle and the segmental mobility.

Variables	One day pre-operation	Two weeks post-operation	Last follow-up
Interspinous distance (mm)	4.2±0.5	9.2±1.1	9.1±1.2
Intervertebral margin heights (mm)			
Anterior margin	13.6±1.5	12.7±1.3	12.9±1.5
Posterior margin	7.7±0.9	11.3±1.4	11.1±1.6
Intervertebral/lumbar foraminal dimensions (mm)			
Width	8.5±1.1	10.8±1.3	10.9±1.4
Height	18.7±2.1	21.4±2.3	21.1±2.5
Segmental lordosis (°)	14.4±1.7	7.5±1.2	7.9±1.4
Segmental mobility (°)	21.6±5.8	6.2±1.6	6.8±1.5

Values are presented as mean ± standard deviation.
